# Comparison between a dual-time-window protocol and other simplified protocols for dynamic total-body ^18^F-FDG PET imaging

**DOI:** 10.1186/s40658-022-00492-w

**Published:** 2022-09-14

**Authors:** Zhenguo Wang, Yaping Wu, Xiaochen Li, Yan Bai, Hongzhao Chen, Jie Ding, Chushu Shen, Zhanli Hu, Dong Liang, Xin Liu, Hairong Zheng, Yongfeng Yang, Yun Zhou, Meiyun Wang, Tao Sun

**Affiliations:** 1 Paul C. Lauterbur Research Center for Biomedical Imaging, Shenzhen Institute of Advanced Technology, Chinese Academy of Sciences, Shenzhen, People’s Republic of China; 2 Henan Provincial People’s Hospital and the People’s Hospital of Zhengzhou, University of Zhengzhou, Zhengzhou, People’s Republic of China; 3 Central Research Institute, United Imaging Healthcare Group Co., Ltd, Shanghai, People’s Republic of China; 4 School of Biomedical Engineering, Shanghai Tech University, Shanghai, People’s Republic of China; 5 United Imaging Research Institute of Innovative Medical Equipment, Shenzhen, People’s Republic of China

**Keywords:** Total-body PET, Simplified protocol, Kinetic modeling, Parametric imaging

## Abstract

**Purpose:**

Efforts have been made both to avoid invasive blood sampling and to shorten the scan duration for dynamic positron emission tomography (PET) imaging. A total-body scanner, such as the uEXPLORER PET/CT, can relieve these challenges through the following features: First, the whole-body coverage allows for noninvasive input function from the aortic arteries; second, with a dramatic increase in sensitivity, image quality can still be maintained at a high level even with a shorter scan duration than usual. We implemented a dual-time-window (DTW) protocol for a dynamic total-body ^18^F-FDG PET scan to obtain multiple kinetic parameters. The DTW protocol was then compared to several other simplified quantification methods for total-body FDG imaging that were proposed for conventional setup.

**Methods:**

The research included 28 patient scans performed on an uEXPLORER PET/CT. By discarding the corresponding data in the middle of the existing full 60-min dynamic scan, the DTW protocol was simulated. Nonlinear fitting was used to estimate the missing data in the interval. The full input function was obtained from 15 subjects using a hybrid approach with a population-based image-derived input function. Quantification was carried out in three areas: the cerebral cortex, muscle, and tumor lesion. Micro- and macro-kinetic parameters for different scan durations were estimated by assuming an irreversible two-tissue compartment model. The visual performance of parametric images and region of interest-based quantification in several parameters were evaluated. Furthermore, simplified quantification methods (DTW, Patlak, fractional uptake ratio [FUR], and standardized uptake value [SUV]) were compared for similarity to the reference net influx rate *K*_*i*_.

**Results:**

*K*_*i*_ and *K*_1_ derived from the DTW protocol showed overall good consistency (*P* < 0.01) with the reference from the 60-min dynamic scan with 10-min early scan and 5-min late scan (*K*_*i*_ correlation: 0.971, 0.990, and 0.990; *K*_1_ correlation: 0.820, 0.940, and 0.975 in the cerebral cortex, muscle, and tumor lesion, respectively). Similar correlationss were found for other micro-parameters. The DTW protocol had the lowest bias relative to standard *K*_*i*_ than any of the quantification methods, followed by FUR and Patlak. SUV had the weakest correlation with *K*_*i*_. The whole-body *K*_*i*_ and *K*_1_ images generated by the DTW protocol were consistent with the reference parametric images.

**Conclusions:**

Using the DTW protocol, the dynamic total-body FDG scan time can be reduced to 15 min while obtaining accurate *K*_*i*_ and *K*_1_ quantification and acceptable visual performance in parametric images. However, the trade-off between quantification accuracy and protocol implementation feasibility must be considered in practice. We recommend that the DTW protocol be used when the clinical task requires reliable visual assessment or quantifying multiple micro-parameters; FUR with a hybrid input function may be a more feasible approach to quantifying regional metabolic rate with a known lesion position or organs of interest.

**Supplementary Information:**

The online version contains supplementary material available at 10.1186/s40658-022-00492-w.

## Introduction

^18^F-FDG positron emission tomography (PET) imaging is widely used for tumor characterization, staging, restaging, and therapy monitoring [1, 2]. Currently, FDG PET quantification is mostly limited to standard uptake value (SUV), a semiquantitative measure derived from static acquisition. Several alternative measures exist to better quantify the differences in subjects, including normalizing the activity concentration by using the lean body mass (SUL) [3] and the body surface area [4], rather than the total body mass. SUV-based measures have a number of disadvantages [5–7]. An optimal scan window, for example, may differ between subjects; inter-study variability in blood supply may also influence quantification; and an uptake image may contain both specific and nonspecific components. To reduce the effect of nonspecific uptake, SUV was proposed to be normalized to the uptake in the background region (SUR) [8] or to the integral of the arterial input function (fractional uptake ratio [FUR]) [9]. To improve the diagnosis, it has also been proposed to assess the relative SUV change between early and late scans [10].

On the other hand, net influx rate *K*_*i*_ is a full-quantitative parameter that outperforms SUV in differentiating malignant from benign lesions and delineating tumor volume [11–16]. Accurate *K*_*i*_ estimation necessitates full dynamic PET acquisition. A standard dynamic acquisition protocol warrants more than 60 min of list-mode acquisition beginning with tracer injection, together with sequential arterial blood sampling. The two most common methods for calculating *K*_*i*_ are irreversible two-tissue compartment model (2T3k) fitting [17] and Patlak graphical analysis [18, 19]. If the arterial input function of the full scan can be approximated, for example, with a population-based one sampled and averaged from previous full dynamic scans, the scan time for Patlak graphical analysis can be significantly reduced [20].

Many efforts have been made to avoid invasive blood sampling and shorten the duration of scans. Several noninvasive methods for determining the input function have been proposed, including image-derived input function (IDIF) [21, 22], population-based input function (PBIF) [23, 24], model-based input function [25], and hybrid input function [26] (combination of IDIF and PBIF). The difficulty in obtaining the input function for a bed position with no large artery in the field of view (FOV) is a concern when using these methods. IDIF derived from carotid artery scanning, for example, is known to be underestimated in brain PET. The FlowMotion technique can obtain the input function for the entire body from the aortic arteries, allowing for whole-body *K*_*i*_ imaging [27]. While for reducing scan time, one option is to replace the long dynamic acquisitions with two static acquisitions, i.e., the dual-time-window protocol. This protocol has been shown to be reliable for obtaining reliable *K*_*i*_ quantification and images [8, 28–30]. The majority of the preceding studies, however, are based on Patlak graphical analysis for FDG, from which only the macro-parameter *K*_i_ (net FDG influx rate) can be obtained. On the other hand, nonlinear estimation based on 2T3k model can also yield micro-kinetic parameters like *K*_1_, *k*_2_, and *k*_3_. Previous research has shown that *K*_1_ is a useful marker for identifying tumor subgroups [31] and assessing chemotherapy response [32]. *k*_3_ was proven to be effective in subtyping of pheochromocytoma and paraganglioma [33]. The combination of *K*_*1*_ and *K*_*i*_ is also useful in gaining access to metabolic tumors [34]. However, a whole-body assessment of these micro-parameters would require a total-body dynamic scan. Nonlinear parametric estimation is known sensitive to the quality of dynamic images; thus, region of interest (ROI)-based kinetic analysis is frequently used instead of voxelized parametric images. One can imagine that the quality of kinetic modeling would be even less reliable with a simplified imaging protocol because the scan data would be less reliable.

Total-body scanners, such as the uEXPLORER or Biograph Vision Quadra, may be able to address these issues in dynamic imaging. First, the whole-body coverage allows for the noninvasive input function from the aortic arteries to be obtained, which has already been shown to be close to the arterial sampling, at least for FDG [35]. Zhang et al. [36] and Sari et al. [37] investigated the feasibility of extracting an input function from dynamic images of the left atrium, left ventricle, aortic artery, and carotid artery, for example. This capability can improve the dependability of IF estimation methods such as PBIF approximation and the hybrid method. Second, with a significant increase in sensitivity, image quality can be maintained at a high level even when scanning for a shorter period of time than usual [38–40]. This will enable the use of more flexible simplified protocols with significantly reduced scan time, allowing for more confident linear or nonlinear parametric estimation. uEXPLORER was used to investigate simplified dynamic FDG total-body imaging protocols. Feng et al. [41] investigated early FDG kinetics using only 2-min post-injection data to obtain whole-body K_1_ images with IDIF. Wu et al. [26] proposed two simplified acquisition protocols for producing whole-body *K*_*i*_ images with hybrid input functions, reducing the scan time to 10 min. Liu et al. [39] obtained 45-min scan data and reported that the quantification of *K*_*i*_ was comparable to the 60-min scan.

In this study, we used a dual-time-window (DTW) total-body FDG scan protocol to obtain multiple kinetic parameters in key areas. The protocol included two dynamic acquisitions: An early acquisition performed 10 min after injection and a late acquisition performed with a fixed end time of 60 min. Nonlinear fitting with rational functions was used to fill the missing data in the interval. The full input function was obtained using a hybrid approach with a population-based image-derived function (PB-IDIF) from 15 subjects. The processed data at each ROI were then used to estimate micro- and macro-kinetic parameters *K*_1_, *k*_2_, *k*_3_, *K*_*i*_, and *V*_*b*_ in 2T3k model. Fast nonlinear least squares fitting at each voxel was also used to generate whole-body parametric images. The proposed DTW protocol quantification was evaluated and compared with existing simplified quantification methods such as SUV, FUR, and Patlak analysis. Finally, we discuss which simplified quantification method is appropriate for specific clinical applications. The novelties of the present work are twofold: First, with 10-min early acquisition and a 5-min late acquisition on a total-body PET, relatively accurate quantifications of *K*_*i*_, *K*_1_, *k*_3_, and *V*_*b*_ at whole-body level were obtained. Second, quantification of DTW was first time compared with several existing simplified quantification methods, including Patlak analysis, FUR, and SUV, as surrogates for the net influx rate *Ki*.

## Materials and methods

### Study subjects and image generation

The present study was approved by the Institutional Review Board of Henan Provincial People’s Hospital, China. After obtaining informed consent, 28 human subjects were enrolled in the study. Table 1 shows the demographic information for all participants. Among the 28 participants, 18 were used for normal organ/tissue quantification, and 10 were used for tumor lesion quantification (one lesion from each). A total-body PET/CT scan was performed on each subject at Henan Provincial People's Hospital using an uEXPLORER PET/CT scanner (United Imaging Healthcare, Shanghai, China). The following are the scan procedures. Following the injection of ^18^F-FDG in the lower extremity vein, a CT scan was performed for attenuation correction, followed by a 60-min list-mode acquisition. The list-mode data were partitioned and reconstructed to produce 66 images (24 × 5 s, 6 × 10 s, 6 × 30 s, 6 × 60 s, 24 × 120 s). Each frame was reconstructed with manufacturer-supplied reconstruction software (TOF-OSEM with 3 iterations and 24 subsets) with the point-spread-function modeled. The reconstruction process compensated for random, scatter, attenuation, normalization, and dead time effects. The reconstructed image had a total of 192 × 192 × 672 voxels. In the transaxial plane, the reconstructed image had a slice thickness of 2.89 mm and a voxel size of 3.125 mm. Visual examinations were performed by an experienced operator to guarantee the enrolled subjects free of visible body motion artifacts. Using frame-by-frame movies, the status of patient movement was inspected in axial, coronal, and sagittal view planes. The exclusion criteria were the positional displacement cannot exceed 3 voxels for several consecutive frames. 3D ROIs were drawn in both normal tissues (cerebral cortex, muscle) and tumor lesions for ROI-based quantifications. The time-activity curve (TAC) at each ROI was created by averaging the activity uptake of all voxels over time and was then used for the quantifications described below. Pixel-by-pixel, whole-body parametric images were also created.

**Table 1:** Demographical information (mean ± s.d.)

	No. of subjects	Age (years)	Gender (male/female)	Height (cm)	Weight (kg)	Injected dose (MBq)
Healthy subjects	18	55.2 ± 5.9	10/8	163.8 ± 8.0	60.9 ± 9.0	227.3 ± 30.9
Subjects with tumor	10	61.5 ± 9.0	6/4	160.6 ± 4.3	57.9 ± 7.4	232.9 ± 44.9

*Five subjects had a malignant tumor in the lung, 4 subjects had a undetermined tumor in the lung, and 1 subject had a malignant tumor in the liver

### Proposed DTW protocol and data processing

**Fig. 1:**
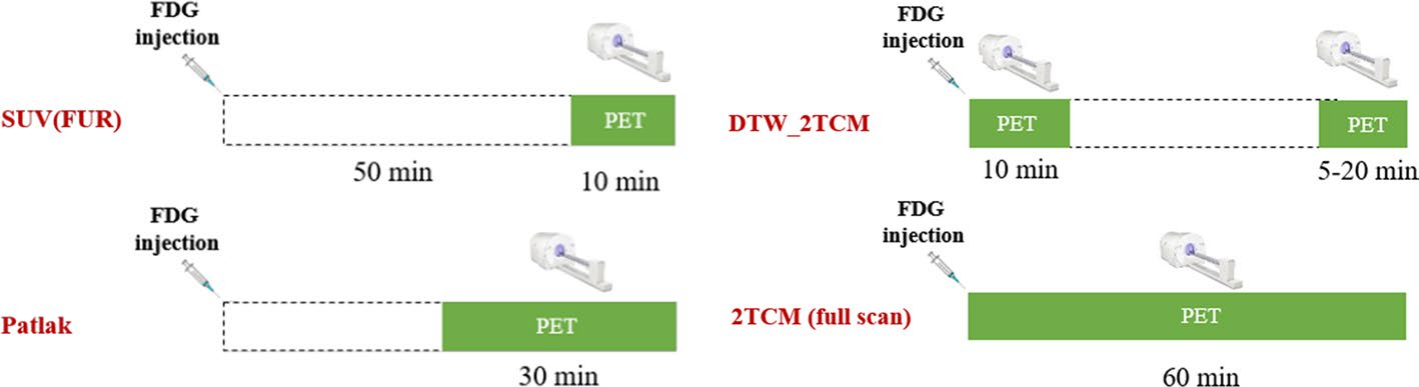
Scan protocols of different quantification methods used in this study

The DTW protocol, as shown in Fig. 1, consists of two short dynamic acquisitions: an early acquisition performed after injection for 10 min and a late acquisition performed after a break with a fixed end time of 60 min. To determine the shortest possible scan time, various late scan durations (5, 10, and 20 min) were tested. DTW (10 + 5 min) refers to the DTW protocol with a 10-min early scan and a 5-min late scan for convenience; similar notations were used for the other two late scan durations. The protocol was tested by discarding the corresponding data in the middle of a 60-min dynamic scan. As a result, the first step in data processing was to fill in the gaps. To estimate the data in the interval, nonlinear fittings to the 3rd degree rational function were used, and the function was as follows:

1 $$C_{E} \left( t \right) = \frac{{p_{1} + p_{3} \times t + p_{5} \times t^{2} + p_{7} \times t^{3} }}{{p_{2} + p_{4} \times t + p_{6} \times t^{2} + p_{8} \times t^{3} }}$$

where *C*_*E*_*(t)* is the fitted tissue activity concentration over time and *p*_1_–*p*_8_ are the parameters that affect the shape of the TAC. (*p*_1_ was fixed to 0, *p*_2_ was fixed to 1, and others were restricted to be positive.) The fitted and the original measured data were then combined to complete the 60-min TACs at each ROI.

Due to the lack of imaging data in the scan interval, the input function was derived using an approach as follows that is similar to the hybrid approach described in the Introduction. From the reconstructed images, IDIF was extracted from the ascending aorta for each subject, where cardiac and respiratory motion was less prominent than at the left ventricle; hence, the partial volume effect (PVE) was less. A 10-mm-diameter ROI was drawn on 6 consecutive slices during early time frames (0–30 s). The hybrid IF was combined with the IDIF from the scan period and scaled with PB-IDIF for the missing part. The PB-IDIF was obtained by averaging the normalized IDIFs of 15 subjects (10 males, 5 females, $53.5 \pm 12.7$ years, $65.7 \pm 9$.2 kg, $249.5 \pm 42.4$ MBq) from the 60-min full dynamic scans. The equation to generate a hybrid IF was as follows:

2 $$C_{P} \left( t \right) = \left\{ {\begin{array}{*{20}c} {C_{{{\text{image}}}} \left( t \right),} & {\left( {0 \le t < t_{1} } \right)} \\ {\mu e^{{ - \gamma \left( {t - t_{1} } \right)}} C_{p0} \left( t \right), } & { \left( {t_{1} \le t < t_{2} } \right)} \\ {C_{{{\text{image}}}} \left( t \right),} & {\left( {t_{2} \le t \le 60 \min } \right)} \\ \end{array} } \right.$$

where *C*_image_*(t)* is from the measured data, *C*_*p0*_*(t)* is the PB-IDIF, t_1_ is the end time of the early scan, t_2_ is the start time of the late scan, and $\mathrm{and }\mu$ and $\gamma$ are the scaling factors. Blood-to-plasma correction was not performed in this study, i.e., Cp(*t*) used the whole blood TAC derived from the images. Delay correction was performed for each ROI as in [42]: The input function was shifted from − 60 to + 60 s, and a 2T3k model was fitted to each regional TAC with the shifted input function. For each region, the delay time that provides the best fitting was thus selected. The dispersion was not considered in the present study. Kinetic modeling was then performed for the complete TAC and input function at each ROI with the 2T3k model. In this model, the dynamic activity change was described by a set of linear ordinary differential equations:

$$\frac{{{\text{d}}C_{1} \left( t \right)}}{{{\text{d}}t}} = K_{1} C_{p} \left( t \right) - \left( {k_{2} + k_{3} } \right)C_{1} \left( t \right) + k_{4} C_{2} \left( t \right)$$

3 $$\frac{{{\text{d}}C_{2} \left( t \right)}}{{{\text{d}}t}} = k_{3} C_{1} \left( t \right) - k_{4} C_{2} \left( t \right)$$

where *C*_*p*_*(t)*, *C*_*1*_*(t)*, and *C*_*2*_*(t)* correspond to activity concentration in plasma, free FDG, and phosphorylated FDG, respectively, and *K*_1_, *k*_2_, *k*_3_, and *k*_4_ denote the transform rates between the compartments. Because the phosphorylation of FDG is considered an irreversible reaction in most tissues, the above-mentioned model was simplified by assuming *k*_4_ = 0. The intensity value of each voxel in a PET image represented the sum of activity concentrations in all compartments:

4 $$C_{{{\text{PET}}}} \left( t \right) = V_{b} C_{b} \left( t \right) + \left( {1 - V_{b} } \right)\left[ {C_{1} \left( t \right) + C_{2} \left( t \right)} \right]$$

where *C*_PET_(*t*) is the measured tissue activity concentration, $C_{b} \left( t \right)$ is the activity concentration in blood, and *V*_*b*_ is the vascular volume fraction. By taking Eq. 3 to Eq. 4, kinetic parameters were estimated by minimizing the objective function:

5 $$\chi^{2} = \mathop \sum \limits_{i = 1}^{N} \omega_{i} \left[ {C_{{{\text{PET}}}} \left( {t_{i} } \right) - C_{t} \left( {t_{i} ,\overline{p}} \right)} \right]^{2}$$

where *C*_*t*_ is the simulated activity concentration by applying the estimated kinetic parameters into Eq. (4), $\overline{p}$ represents *K*_1_, *k*_2_, *k*_3_, and *V*_*b*_; *i* is the frame index; and ${\omega }_{i}$ is the weight at each frame.

Apart from the ROI-based analysis, we also performed a voxel-based analysis aiming to generate parametric images for each simplified dynamic scan. Given the large number of voxels in a whole-body image, the conventional nonlinear problem in Eq. 3 was reformed into a linearized problem [43, 44], as shown in the following equation:

6 $$C_{T} (t) = P_{1} C_{P} (t) + P_{2} \int_{0}^{t} {C_{P} (\tau )} {\text{d}}\tau + P_{3} \int_{0}^{t} {\int_{0}^{\tau } {C_{P} (s){\text{d}}s{\text{d}}\tau } } + P_{4} \int_{0}^{t} {C_{T} (\tau )} {\text{d}}\tau + P_{5} \int_{0}^{t} {\int_{0}^{\tau } {C_{T} (s){\text{d}}s{\text{d}}\tau } }$$

where *P*_1_, *P*_2_, *P*_3_, *P*_4_, and *P*_5_ are the functions of kinetic parameters to be estimated. Lawson–Hanson NNLS (nonnegative least squares) algorithm was then applied to solve the above equation. This will accelerate the estimation dramatically, thereby allowing the generation of whole-body multiparametric images within 1 min in our experience. After obtaining *K*_1_, *k*_2_, and *k*_3_, one can compute the macro-kinetic parameter (image) *K*_*i*_:

7 $$K_{i} = \frac{{K_{1} \times k_{3} }}{{k_{2} + k_{3} }}$$

which represents the net FDG influx rate and is a surrogate to the glucose influx rate. The unit for *K*_*i*_ and *K*_1_ is ml/g/min, while for *k*_2_ and *k*_3_ is min^−1^. In this study, Lawson–Hanson NNLS was used to generate multiparametric (*K*_1_, *k*_2_, *k*_3_, *V*_*b*_, *K*_*i*_) images using full dynamic data, and Patlak *K*_*i*_ images using 30-min data were generated for comparison.

### Other simplified quantification methods

*Standardized uptake value (SUV):* SUV was calculated using the following equation:

8 $${\text{SUV}} = \frac{{C_{{{\text{PET}}}} \left( T \right)}}{A/W}$$

where C_PET_(T) is the mean activity concentration in 50–60 min, and *A* and *W* are the total injected activity (Bq/cc) and body weight (kg) of the subject, respectively.

*Fractional uptake ratio (FUR):* FUR is defined as in [9, 45]:

9 $${\text{FUR}} = \frac{{C_{{{\text{PET}}}} \left( T \right)}}{{\mathop \smallint \nolimits_{0}^{T} C_{p} \left( t \right){\text{d}}t}}$$

where *C*_PET_(*T*) is normalized by the integral of plasma activity concentration from the start to time *T*. Like SUV, FUR can be calculated from a single static PET scan (50–60 min in this study). However, the input function is still required to calculate the integral in the denominator. Similar to the proposed protocol, a hybrid input function was used here with a different method of scaling:

10 $$C_{p} \left( t \right) = \left\{ {\begin{array}{*{20}c} {\alpha \cdot C_{p0} \left( t \right), \left( {0 \le t < 50 \min } \right)} \\ {C_{{{\text{PET}}}} \left( t \right), \left( {50 \le t \le 60 \min } \right)} \\ \end{array} } \right.$$

where the area under the curve (AUC) in 50–60 min was used to scale PB-IDIF *C*_*p*0_(*t*) with a factor *α* to obtain the full input function.

*Patlak graphical analysis:* Patlak analysis [18, 19] can be expressed as a linear regression process:

11 $$\frac{{C_{{{\text{PET}}}} \left( T \right)}}{{C_{p} \left( T \right)}} = K_{i} \times \frac{{\mathop \smallint \nolimits_{0}^{T} C_{p} \left( t \right){\text{d}}t}}{{C_{p} \left( T \right)}} + {\text{int}} \left( {t \ge t^{*} } \right)$$

where *C*_PET_(*T*) and *C*_*p*_(*T*) are the activity concentration in tissue and plasma at time *T*(0–60 min), respectively; *K*_*i*_ denotes the net FDG influx rate; int is the y-axis intercept of the regression plot; and ${t}^{*}$ is the time point (30 min in this study) when an equilibrium between blood and tissue is reached. The input function for Patlak analysis was obtained in the same manner as that for FUR.

### Statistical analysis

All statistical analyses were performed using Statistical and Machine Learning Toolbox in MATLAB R2018b. Twelve subjects (5 males, 7 females, 57.8 ± 9.1 years, 62.5 ± 12.3 kg, injection dose $230.3 \pm 47.6$ MBq) were randomly chosen from the subjects listed in Table 1 to evaluate the comparability of the hybrid IFs to IDIFs with correlation analysis. To verify the accuracy of the complete TAC, the mean absolute percentage error (MAPE) of all time points in the interval was calculated for each subject:

12 $${\text{MAPE}}\_{\text{TAC}}\left( \% \right) = \frac{{\mathop \sum \nolimits_{i} \left| {C_{E} \left( {t_{i} } \right) - C_{M} \left( {t_{i} } \right)} \right|/C_{M} \left( t \right)}}{n} \times 100\%$$

where i is the index of the frame in the interval; *t*_*i*_ is the sample time point; *n* is the total number of sample time points; and *C*_*E*_ and *C*_*M*_ are the estimation and true measured activity concentrations, respectively. The Bland–Altman distribution of the MAPE in TAC estimation for each ROI was plotted. The parameters computed by 2T3k from the entire 60-min scan were used as the reference for the ROI-based quantification. For each ROI, the kinetic parameters (*K*_1_, *k*_2_, *k*_3_, *V*_*b*_, and *K*_*i*_) were calculated and compared to the reference. Correlation determination and bias analysis were carried out for various scan duration configurations. The percentage bias of *K*_*i*_ and *K*_1_ from the DTW protocol was calculated as:

13 $${\text{Bias}}\_K\left( \% \right) = \frac{{K_{{{\text{ref}}}} - K_{{{\text{DTW}}}} }}{{K_{{{\text{ref}}}} }} \times 100\%$$

where $K_{{{\text{ref}}}}$ and $K_{{{\text{DTW}}}}$ denote a specific parameter from the standard full scan protocol and the DTW protocol, respectively. Correlation analysis was performed for each simplified quantification method to the true quantification from the full scan, where *P* < 0.05 was considered as a significant correlation.

## Results

### Hybrid IFs and TAC completion

**Fig. 2:**
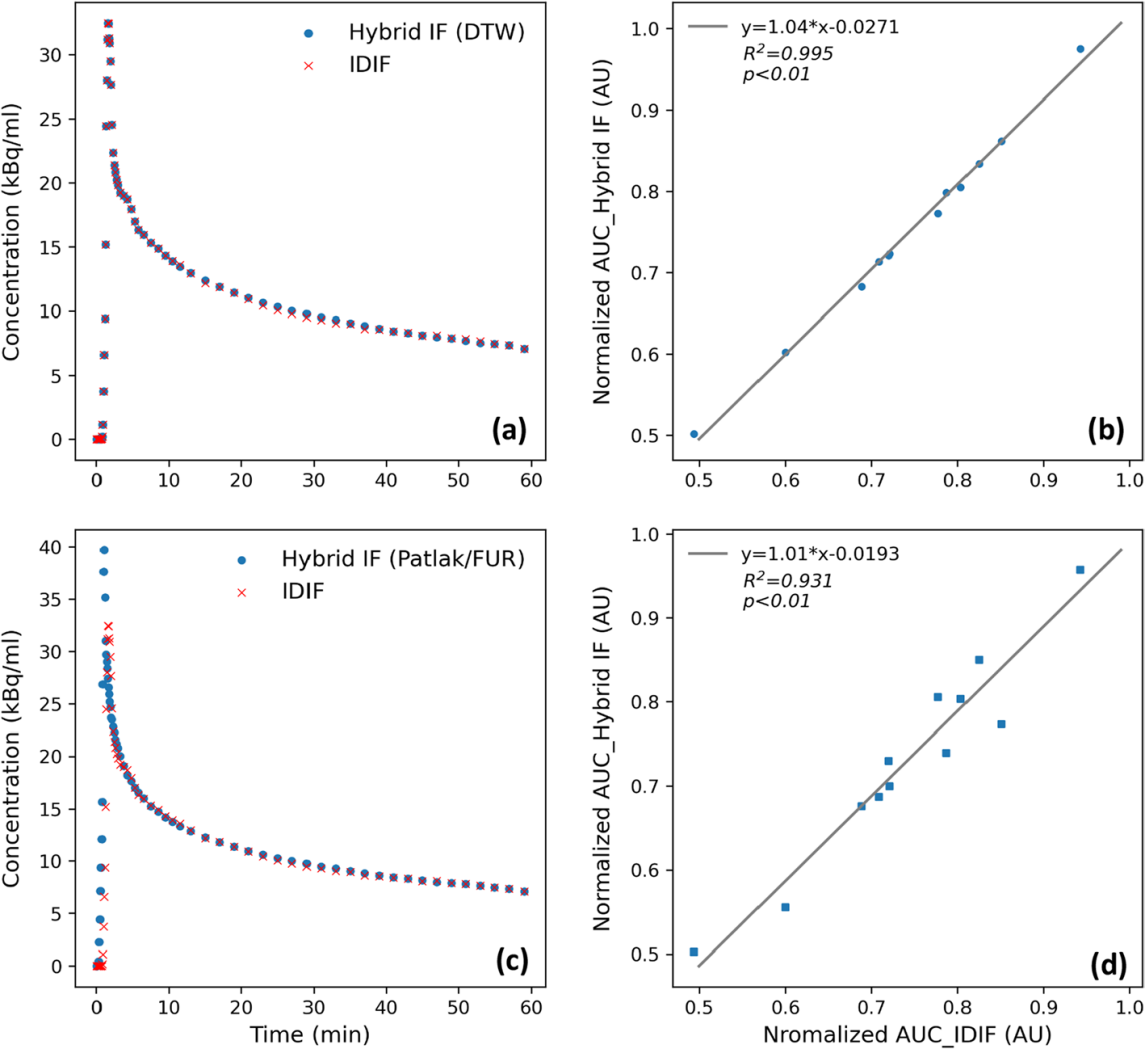
Example of hybrid IFs used for DTW (**a**) and Patlak/FUR quantifications (**c**). Their corresponding correlations to the true IDIFs are shown in (**b**) and (**d**), respectively

The IFs for both DTW and FUR/Patlak analysis were generated according to the steps described in Methods section. Correlation of AUCs between the hybrid IFs and the reference IDIFs from the full scan was determined. As shown in Fig. 2, relatively high correlations were observed for both DTW (*R*^2^ = 0.995, *y* = 1.04*x-0.0271) and FUR/Patlak (*R*^2^=0.931, *y* = 1.01*x-0.0193).

**Fig. 3:**
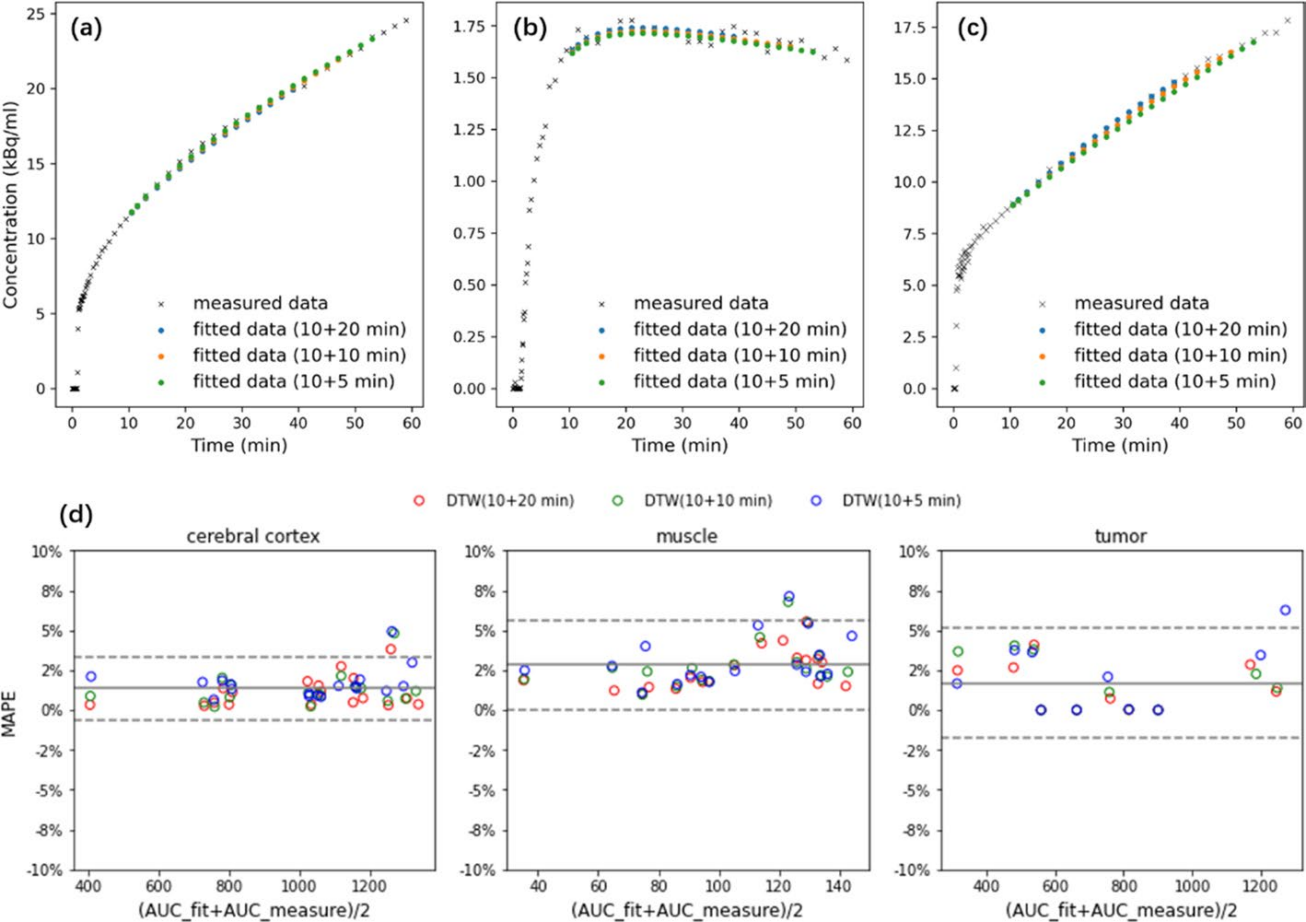
For an example subject, nonlinear fitting of the missing data was performed using the 3rd degree rational function. **a**–**c** correspond to the TACs in the cerebral cortex, muscle, and tumor, respectively; **d** Bland–Altman plots of the MAPE (mean absolute percentage error, Eq. 12) in TAC estimation for each ROI with different scan durations

The estimated TACs in ROIs were compared to the reference measured ones from a full scan. Figure 3d shows the distribution of the MAPE for each ROI. Even for the protocol with the shortest scan duration (a 10-min early scan and a 5-min late scan), the TACs after completion were generally close to the reference ones. It is true, however, that estimation accuracy decreases as scan time decreases.

### ROI-based quantification for the DTW protocol

**Fig. 4:**
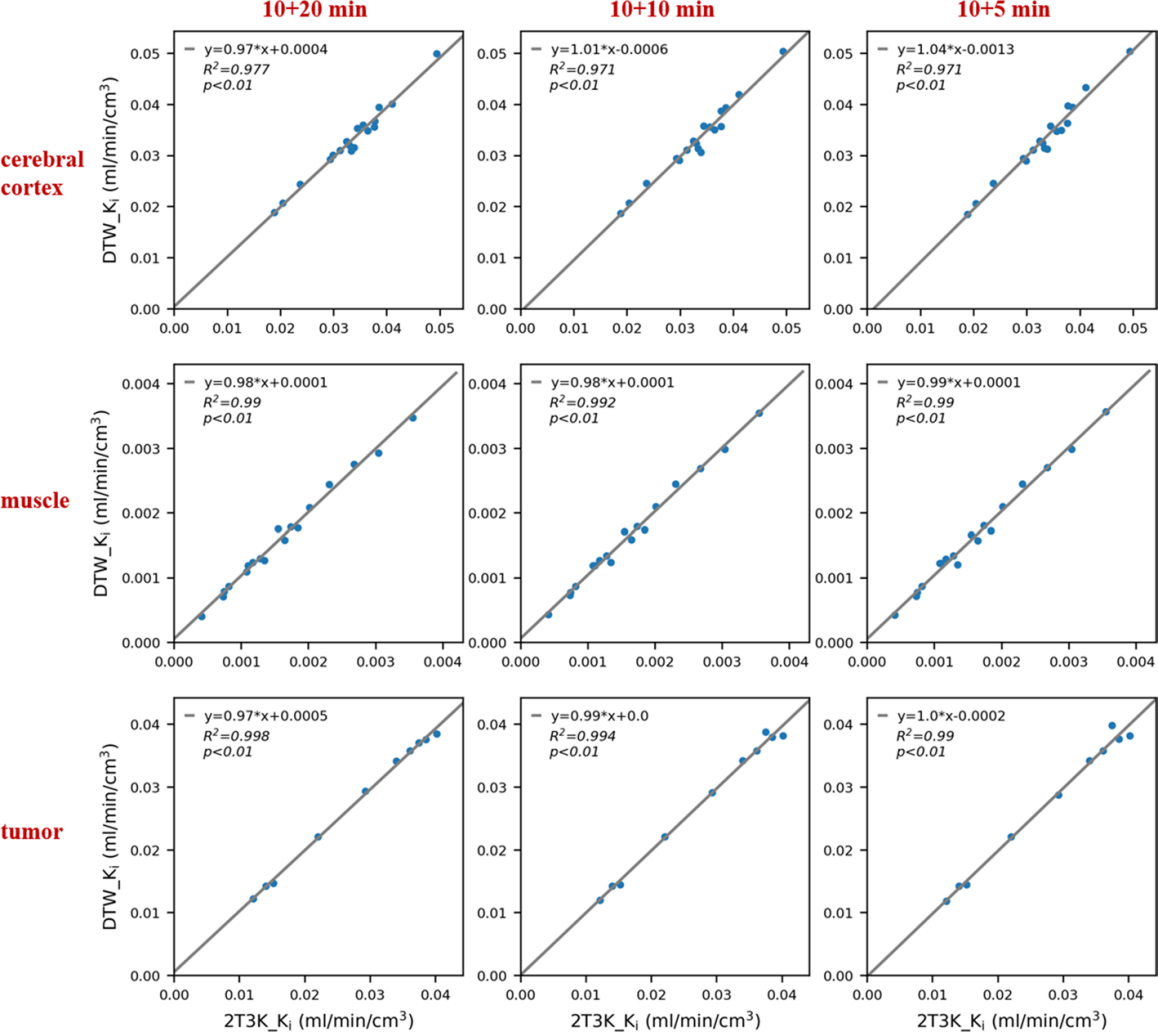
Correlation analysis for *K*_*i*_ derived from the DTW protocol with different scan durations. The ROIs were sampled in the cerebral cortex, muscle, and tumor lesion. The correlation plot for *K*_1_ is shown in Additional file 1: Fig. S1. The associated Bland–Altman plots are shown in Additional file 1: Fig. S5

**Fig. 5:**
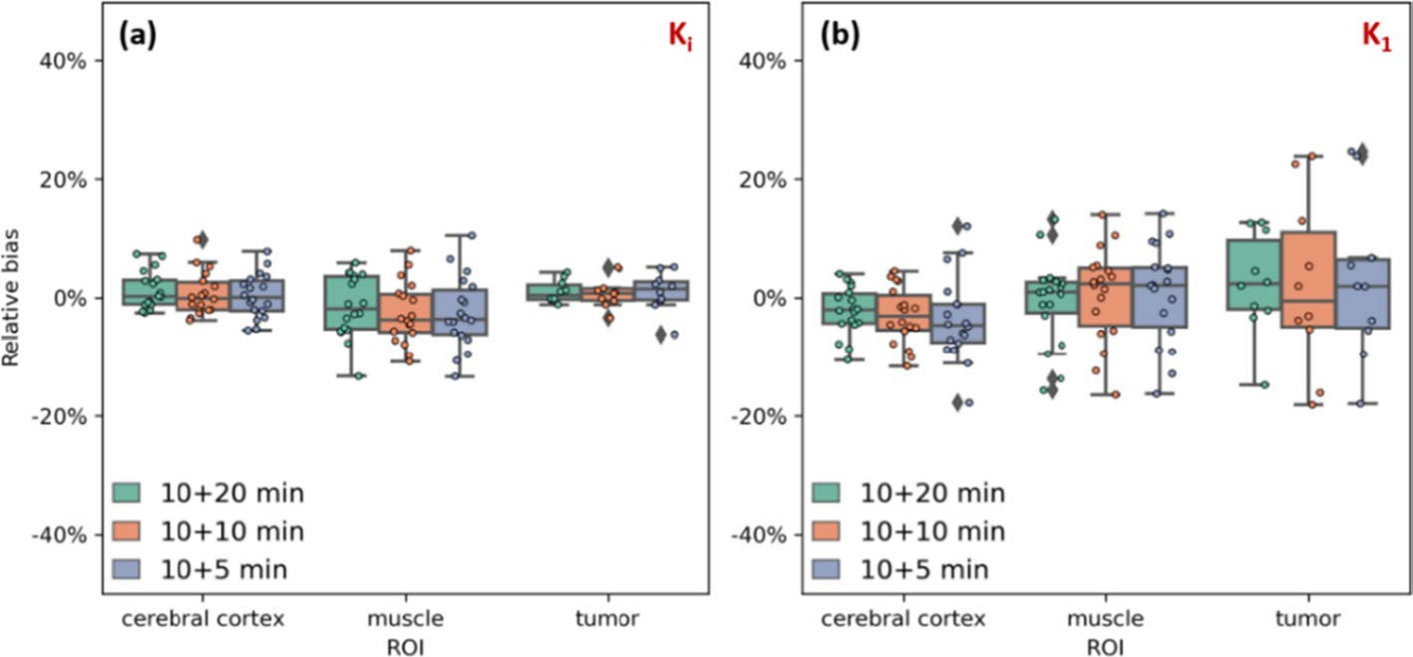
Distribution of the percentage bias in *K*_*i*_ (**a**) and *K*_1_ (**b**) derived from the DTW protocol

The correlation results of *K*_*i*_ are shown in Fig. 4. In general, as the late scan duration decreased, the correlation also decreased. Nonetheless, even for the 5-min late scan, a relatively high correlation was observed, with correlation coefficients and linear regression equation being (0.971, *y* = 1.04*x − 0.0013), (0.990, *y* = 0.990*x + 0.0001), and (0.990, *y* = x − 0.0002) for the cerebral cortex, muscle, and tumor lesion, respectively. Figure 5a depicts the corresponding percentage bias distributions in *K*_*i*_. The bias varied across the ROIs, with the cerebral cortex, muscle, and tumor lesion being − 0.1 ± 3.2%, − 3.3 ± 6.7%, 1.2 ± 2.5%, respectively. As shown in Additional file 1: Fig. S1, the correlation coefficients and linear regression equations of *K*_1_ derived from DTW (10 + 5 min) comparing with the reference were (0.820, *y* = 1.03*x + 0.0006), (0.940, *y* = 0.890*x + 0.0028), and (0.975, *y* = 0.98*x + 0.0018) for the cerebral cortex, muscle, and tumor lesion, respectively. Figure 5b depicts the bias analysis for *K*_1_. The bias of *K*_1_ in estimation varied across ROIs, with the cerebral cortex, muscle, and tumor being 5.2 ± 8.6%, 2.6 ± 9.1%, 1.7 ± 7.9%. In all regions, the bias in *K*_1_ was greater than that in *K*_*i*_. In Additional file 1: Figs. S2 and S3 show the correlation analysis for *k*_3_ and *V*_*b*_.

### Comparison among different simplified quantification methods

The proposed protocol's quantification was compared to three existing FDG quantification methods, namely SUV, FUR, and Patlak analysis. The *K*_*i*_ estimated from a full dynamic scan was used as a reference. Table 2 displays *K*_*i*_ correlations. The scattering plots for these correlations are shown in Additional file 1: Fig. S4. The DTW protocol (10 + 5 min) had the highest quantification accuracy relative to the reference *K*_*i*_ of all the simplified quantification methods, followed by FUR and Patlak analysis. In all three sampled regions, SUV had the lowest correlation.

**Table 2:** Correlation analysis for *K*_*i*_ and its surrogate parameters from different quantification methods; the squared correlation coefficient (*R*^2^), *p* value(*p*), and linear regression equations with each quantification method were listed in the table and the results of strongest correlation was marked in bold

Methods	Cerebral cortex	Muscle	Tumor
DTW K_i_ (10 + 5 min)	*R*^2^ = **0.971**	*R*^2^ = **0.990**	*R*^2^ =** 0.990**
	*p* < 0.01	*p* < 0.01	*p* < 0.01
	*y* = 1.04*x − 0.0013	*y* = 0.099*x + 0.0001	*y* = *x *− 0.0002
Patlak K_i_ (30–60 min)	*R*^2^ = 0.961	*R*^2^ = 0.863	*R*^2^ = 0.990
	*p* < 0.01	*p* < 0.01	*p* < 0.01
	*y* = 0.99*x + 0.002	*y* = 0.94*x + 0.0002	*y* = 0.99*x + 0.0008
FUR (50–60 min)	*R*^2^ = 0.967	*R*^2^ = 0.937	*R*^2^ = 0.972
	*p* < 0.01	*p* < 0.01	*p* < 0.01
	*y* = 1.13*x + 0.0033	*y* = 1.2*x + 0.0013	*y* = 1.08*x + 0.0028
SUV (50–60 min)	*R*^2^ = 0.447	*R*^2^ = 0.729	*R*^2^ = 0.390
	*p* < 0.01	*p* < 0.01	*p* = 0.054
	*y* = 126.66*x + 1.722	*y* = 159.22*x + 0.2178	*y* = 121.1*x + 1.113

**Fig. 6:**
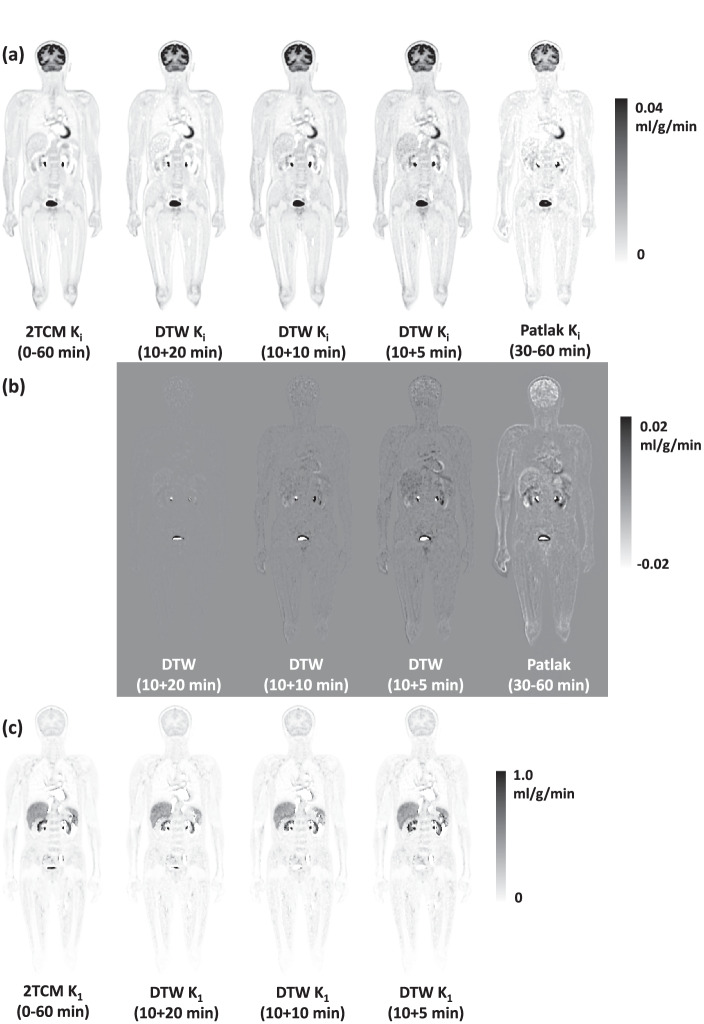
Whole-body parametric images from a dynamic scan: (**a**) *K*_*i*_ images generated from the 60-min full scan, the DTW protocols, and Patlak analysis; the muscle region inside the red box was sampled to quantify the image noise; (**b**) difference images between the ones and the reference *K*_*i*_ image from 60-min full scan; (**c**) *K*_1_ images generated from the 60-min full scan and the DTW protocols;

The results for the visual appearance of the parametric images were overall consistent with the ROI-based quantifications. Figure 6a shows the example *K*_*i*_ images generated by the reference 60-min full scan, the DTW protocols, and Patlak analysis; Fig. 6b shows the difference images between the *K*_*i*_ derived from DTW protocols or Patlak analysis and the reference *K*_*i*_ images. It can be seen that the bias of *K*_*i*_ increases as the duration of the DTW protocol decreases; however, even with a 15-min total scan time, the *K*_*i*_ image is still more consistent with the reference *K*_*i*_ image than the one generated by Patlak analysis. The coefficient of variation in sampled muscle region (red box in Fig. 6a) was used to quantify the noise level of *K*_*i*_ images. For the reference *K*_*i*_ images, the mean and standard deviation of the coefficient of variation are 0.21 ± 0.003; for *K*_*i*_ images derived from DTW protocols, the values are 0.24 ± 0.005 (10 + 20 min), 0.28 ± 0.005 (10 + 10 min), 0.31 ± 0.007 (10 + 5 min), respectively; and for Patlak *K*_*i*_ images, the value is 0.83 ± 0.18, which is higher than others and indicated an overall higher level of noise. *K*_1_ images derived from the DTW protocols were almost visually identical to the reference, as shown in Fig. 6c.

## Discussion

The DTW FDG scan protocol was implemented on a state-of-the-art uEXPLORER scanner in this study, and multiple kinetic parameters were quantified. Whole-body imaging allows for a simultaneous scan of the entire human body with unprecedented sensitivity, as well as the extraction of IDIF directly from large arteries such as the aorta. Multiple kinetic parameters can be obtained by using the processed TACs and the input function at the designated organs. The results showed that with a 10-min early acquisition and a 5-min late acquisition, relatively accurate quantifications of *K*_*i*_, *k*_1_, *k*_3_, and *V*_*b*_ could be obtained. The estimation bias could be reduced with a longer scan duration. Furthermore, we compared the quantification of DTW to several existing simplified quantification methods, including Patlak analysis, FUR, and SUV, as a surrogate for the net influx rate *K*_*i*_. The proposed DTW protocol produced the most accurate quantification, followed by FUR and Patlak analysis, while SUV had the lowest correlation, which is consistent with previous study findings [20, 26, 46]. In terms of visual performance, the DTW protocol generated parametric *K*_*i*_ and *K*_1_ images that were more consistent with the reference and had less noise than the Patlak analysis had.

To reduce the total scan time while maintaining an acceptable quantification accuracy, nonlinear estimation was applied to the shortened dynamic scan data. DTW protocol allows for the efficient use of hospital facilities while maintaining quantification accuracy. For example, interleaved scans become possible, during which the first scan of the second patient can be acquired, while the first patient rests outside the scanning room. The potential inaccuracy of the estimated IFs and TACs is a disadvantage of this method. However, it was found that combining the scaled PB-IDIF with the existing part IDIF from the dual-phase scan can provide accurate IF. TACs estimated using nonlinear fitting with a rational function were also close to the true ones. Inferring parameters from reduced scan data did introduce bias in *K*_*i*_ and *K*_1_ estimation. Using the tumor lesion as an example, *K*_*i*_ estimation was quite accurate with a relative bias of less than 5%, while *K*_1_ estimation had a relative bias of less than 20%. One possible explanation is that the size of some tumors is small and hence TACs are quite unstable compared to uniform regions such as a muscle. This is especially true for the early stage of the scan, where estimation of *K*_1_ depends mostly on. Another possibility is that some tumors had not reached true equilibrium even after 60 min of scanning. Estimation based on 2T3k may not be optimal in such cases. Regarding the nonlinear fitting to complete the missing data in the interval, it took 1 min to run over a single dynamic scan.

There are some limitations to the current study: (1) A direct comparison between the hybrid input function and the arterial sampled input function is missing. (2) Although the primary goal of this work is to compare against DTW to existing simplified measures, it may be an unfair comparison since they have different amount of data available for kinetic modeling. For instance, Patlak, FUR, SUV all use single scan. DTW protocol with more scan information in theory can achieve more accurate estimation in kinetic parameters. In the future, it may be necessary to compare the current DTW protocol with other published dual-time-window methods, such as dual-time-point Patlak analysis [29] and retention index [46]. (3) A 60-min scan may not be long enough to capture the kinetics in some organs, such as the brain and some malignant tumors, to reach the equilibrium state. To avoid the potential bias in *K*_*i*_ and *K*_1_ quantification caused by this factor, a longer scan time may be required. (4) The dispersion effect for the input function was not modeled by our method. This could have resulted in quantitative inaccuracies in head and neck organ measurements [47]. Furthermore, scatter corrections may be insufficiently accurate due to the very concentrated and rapidly changing tracer distribution in the first few seconds after injection. (5) There could be a misalignment in patient position between the early and late phases of the DTW protocol’s actual implementation that affects the accuracy of the kinetic parameter estimation. It may be necessary to align and perform new reconstructions with the re-aligned attenuation image. To avoid two attenuation CT scans, one option is to use the TOF information (e.g., MLAA [48]) or deep learning techniques [49]) to generate the pseudo-attenuation map for the second scan. Then, one can register the first CT image to this attenuation map and hence apply the transformation to the corresponding PET data. Low-dose CT could be another solution to reduce the total amount of radiation exposure, which will require dedicated denoising techniques such as deep learning. (6) The current DTW protocol was implemented on a total-body scanner, which is available in few institutions for now. In theory, it can also be applied on a conventional scanner with short FOV. In such case, full kinetic analysis can only be performed near the chest region since conventional scanner need to capture the input function from the early dynamics of the descending aorta. Therefore, at least the micro-parameters cannot be obtained for non-chest regions. Another potential issue when applying DTW protocol on a conventional scanner would be the relatively lower sensitivity compared with the uEXPLORER which could hamper the estimation from the partial scan data, especially when assessing voxelized parametric images. (7) The number of patients included in the study is limited, given the difficult in acquiring a dynamic scan without patient motion affected. A dynamic total-body scan may be vulnerable to patient movement, especially when considering the increased sensitivity. Motion could potentially affect not only the visual quality but also the accurate quantification across the entire body. In this work, we only performed the simple visual inspection to exclude the datasets with obvious motion artifacts. Therefore, an even more thorough quantitative quality control on the effect of motion may be required other than simply checking visually the images frame by frame. In case when motion is severe, motion compensation [50–52] will be essential before applying the data analysis to a given dynamic scan.

The study’s primary goal was to investigate and improve the feasibility of using the total-body dynamic imaging protocol in clinics. Although a total-body scanner greatly aids in this goal, we believe that not all applications are suitable for dynamic imaging, even with proper scan time reduction. When weighing the benefits of increased absolute quantification accuracy versus the additional effort required, some existing alternatives to SUV may be preferable. As demonstrated by the findings, when the clinical task is to quantify the regional metabolic rate for a known lesion position or organs of interest, FUR with the hybrid IF is the most feasible protocol because it requires a regular scan time with an acceptable bias in *K*_*i*_ estimation. However, when detecting an unknown lesion that necessitates a reliable visual assessment or quantifying micro-kinetic parameters such as *K*_1_, the DTW protocol may be preferable. It would be also interesting to investigate which simplified quantification should be used for applications such as assessing the treatment response. More research for specific applications should be done to minimize the trade-off between accuracy achieved and the extra effort required for dynamic imaging.

## Conclusion

In this study, we evaluated a DTW protocol for FDG quantification and compared its accuracy to that of existing simplified quantification methods, e.g., Patlak analysis, FUR, and SUV. The results showed that by using the DTW protocol, the dynamic total-body FDG scan time could be reduced to 15 min. It is possible to achieve accurate *K*_*i*_ and *K*_1_ quantification as well as acceptable visual quality in parametric images. We anticipate seeing benefits from applying a similar concept to dynamic imaging with other radiotracers, such as ^68^ Ga-PSMA. Although scanners such as uEXPLORER can help to shorten the scan time, more time and effort are still required when compared to traditional SUV static imaging. As a result, other existing simplified quantifications, such as FUR or SUR, may be more appropriate at least in certain applications.

## Supplementary Information

**Additional file 1.** **Fig. S1-S3:** Correlation analysis between micro-parameters (*K*_*1*_, *k*_*3*_, *Vb*) derived from the proposed DTW protocol and  reference values. **Fig. S4:** Correlation analysis between all simplified quantification methods - DTW (10+5 min), Patlak *K*_*i*_, FUR and SUV to the reference *K*_*i*_ in all ROIs. **Fig. S5:** Bland-Altman plots of the percentage bias in *K*_*i*_ and *K*_*1*_ derived from the DTW protocol. **Fig. S6:** Correlation analysis between *K*_*i*_ estimated by non-linear fitting and Lawson-Hanson NNLS fitting.

## Data Availability

The data and material will be available upon reasonable request.

## Abbreviations

PET: Positron emission tomography
CT: Computerized tomography
FDG: Fluorodeoxyglucose
SUV: Standardized uptake value
FUR: Fractional uptake ratio
TOF: Time of flight
OSEM: Ordered subset expectation–maximization
2T3k: Irreversible 2-tissue compartment model
DTW: Dual time window
IDIF: Image-derived input function
PBIF: Population-based input function
PB-IDIF: Population-based image-derived input function
TAC: Time activity curve
ROI: Region of interest
PVE: Partial volume effect
