# Supplementary material for: Comparison between a dual-time-window protocol and other simplified protocols for dynamic total-body 18F-FDG PET imaging
Source: EJNMMI Phys. 2022 Sep 14;9:63. doi: 10.1186/s40658-022-00492-w (PMC9474964; doi:10.1186/s40658-022-00492-w)
Supplement: Supplementary file 1 — Additional file 1. Fig. S1-S3: Correlation analysis between micro-parameters (K1, k3, Vb) derived from the proposed DTW protocol and reference values. Fig. S4: Correlation analysis between all simplified quantification methods - DTW (10+5 min), Patlak Ki, FUR and SUV to the reference Ki in all ROIs. Fig. S5: Bland-Altman plots of the percentage bias in Ki and K1 derived from the DTW protocol. Fig. S6: Correlation analysis between Ki estimated by non-linear fitting and Lawson-Hanson NNLS fitting. [file 40658_2022_492_MOESM1_ESM.docx]

**Supplemental Information File**

**Comparison between a dual-time-window protocol and other simplified protocols for dynamic total-body ^18^F-FDG PET imaging**

Zhenguo Wang^1^, Yaping Wu^2^, Xiaochen Li^2^, Yan Bai^2^, Hongzhao Chen^1^, Jie Ding^1^, Chushu Shen^1^, Zhanli Hu^1^, Dong Liang^1^, Xin Liu^1^, Hairong Zheng^1^, Yongfeng Yang^1^, Yun Zhou^3,4^, Meiyun Wang^2^*, Tao Sun^1,5^*

^1^Paul C. Lauterbur Research Center for Biomedical Imaging, Shenzhen Institute of Advanced Technology, Chinese Academy of Sciences, Shenzhen, People's Republic of China

^2^Henan Provincial People’s Hospital and the People’s Hospital of Zhengzhou, University of Zhengzhou, People's Republic of China

^3^Central Research Institute, United Imaging Healthcare Group Co., Ltd, Shanghai, People's Republic of China

^4^School of Biomedical Engineering, Shanghai Tech University, Shanghai, People's Republic of China

^5^United Imaging Research Institute of Innovative Medical Equipment, Shenzhen, People's Republic of China

*Corresponding Author

Tao Sun, PhD, tao.sun@siat.ac.cn

Meiyun Wang, PhD, mywang@ha.edu.cn


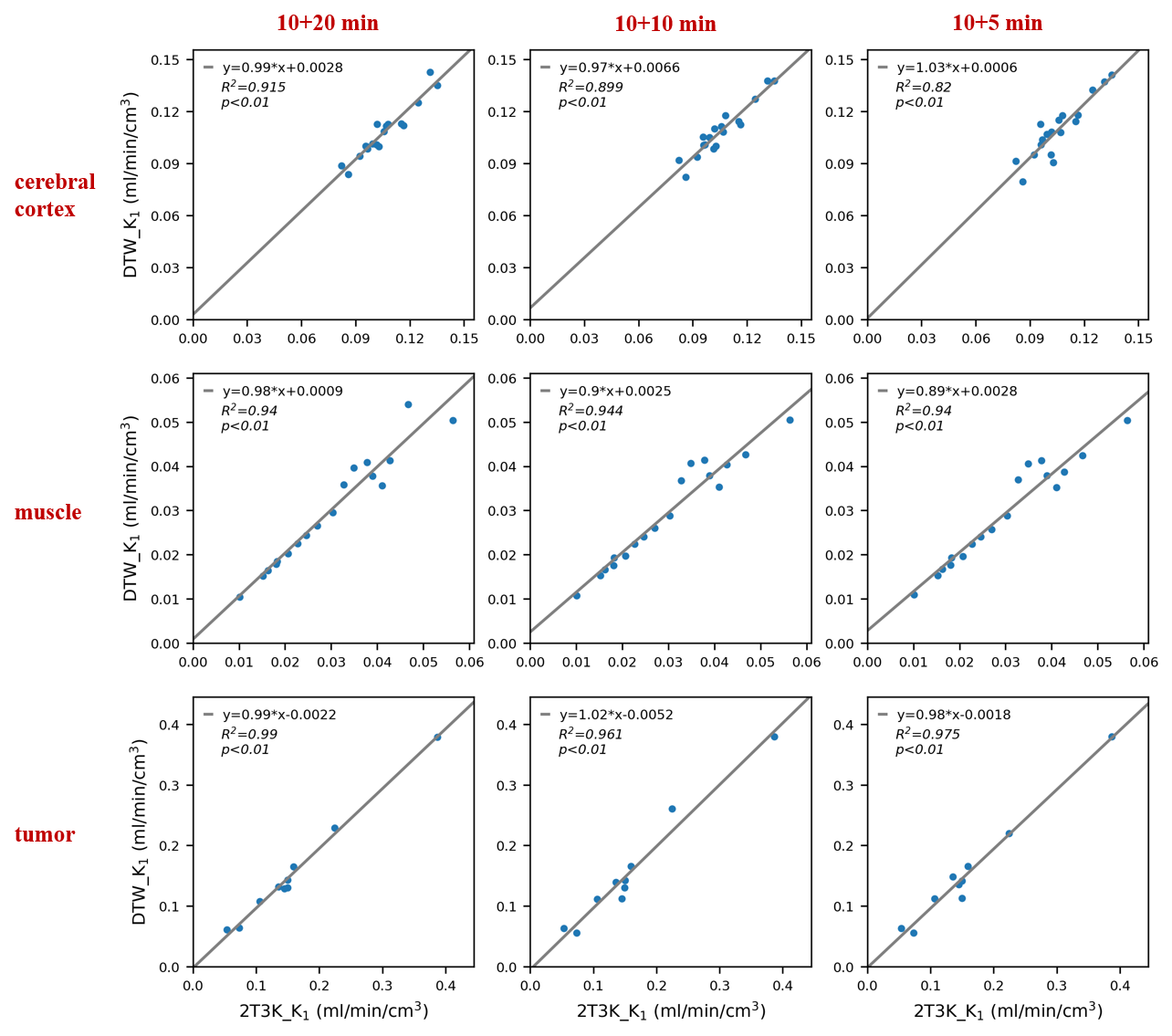


Supplement Figure 1. Correlation analysis between K_1_ derived from DTW protocol with different scan durations and the one from the reference in each ROI sampled in cerebral cortex, muscle and lesion.


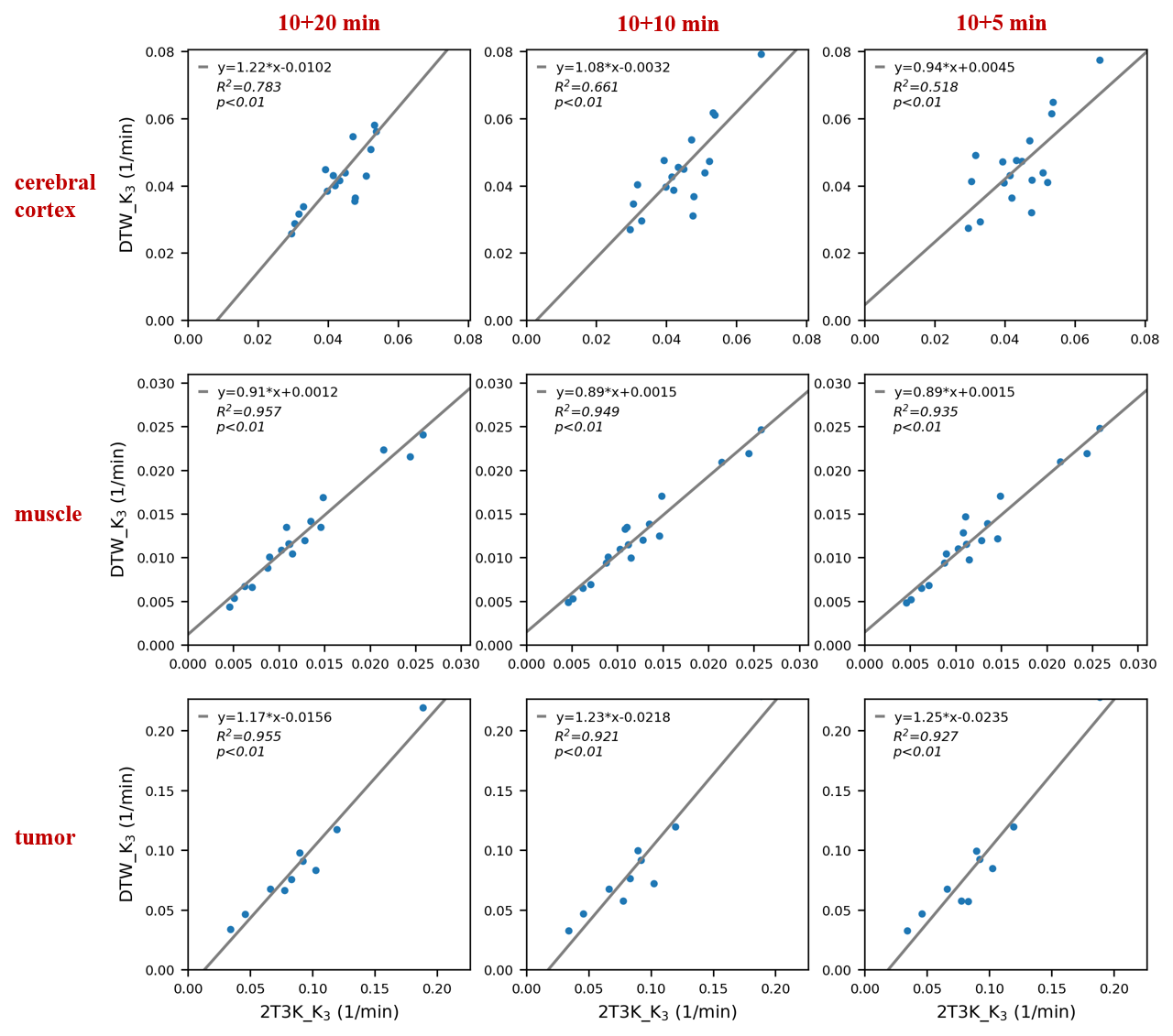


Supplement Figure 2. Correlation analysis between k_3_ derived from DTW protocol with different scan durations and the one from the reference in each ROI sampled in cerebral cortex, muscle and lesion.


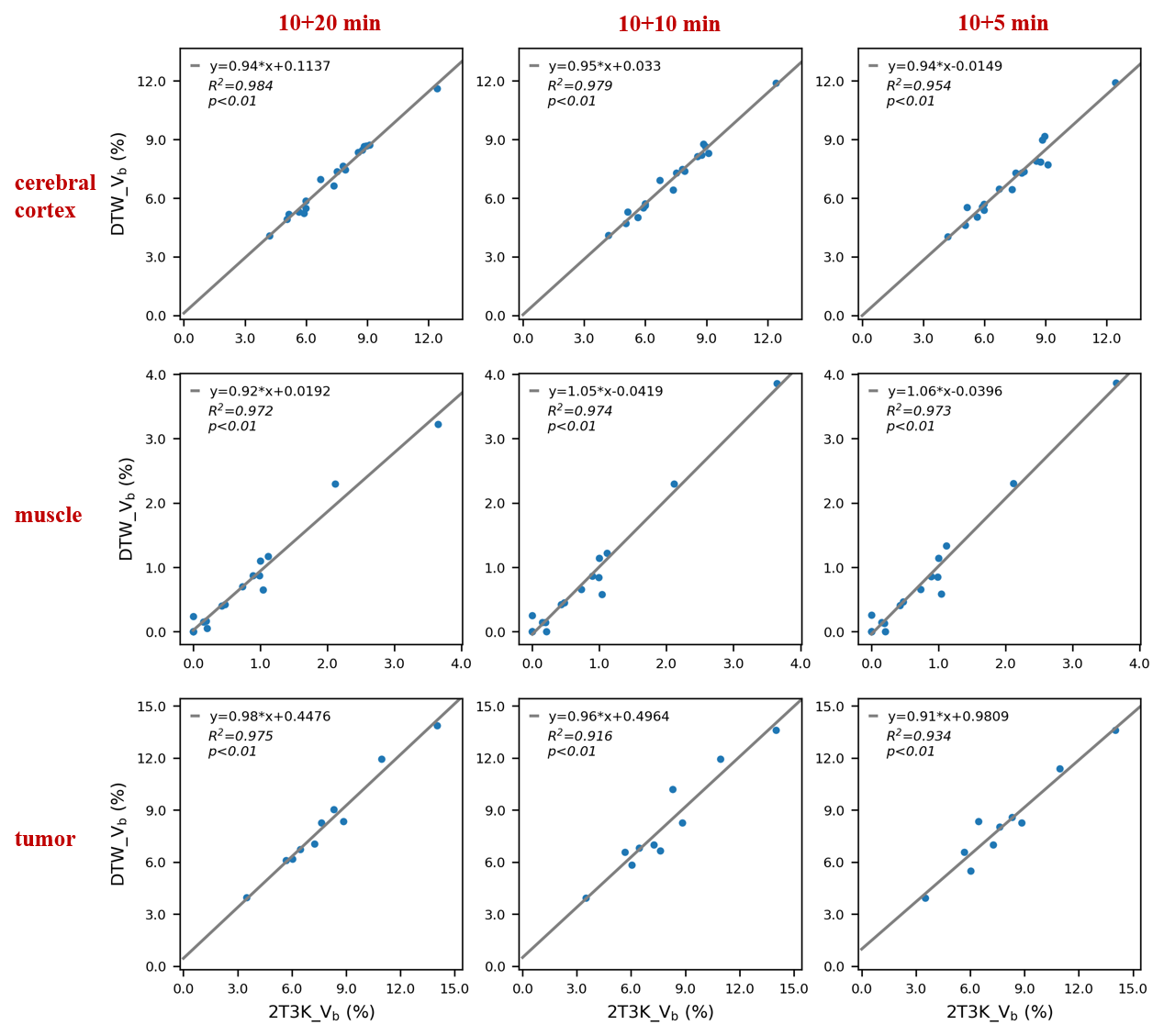


Supplement Figure 3. Correlation analysis between V_b_ derived from DTW protocol with different scan durations and the one from the reference in each ROI sampled in cerebral cortex, muscle and lesion.


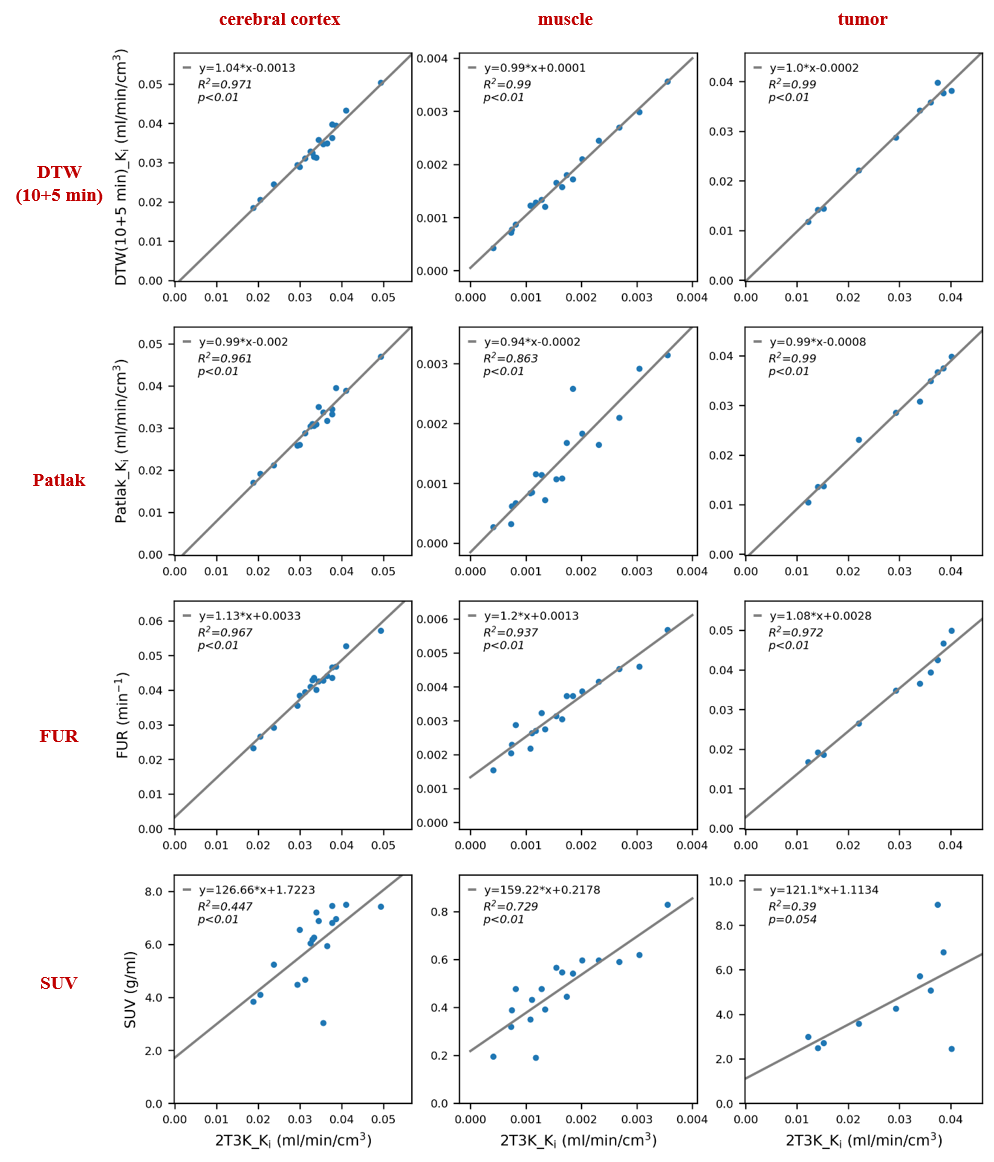


Supplement Figure 4 Correlation analysis between all simplified quantification methods DTW (10+5 min) Patlak K_i_, FUR and SUV to the reference K_i_ in all ROIs.


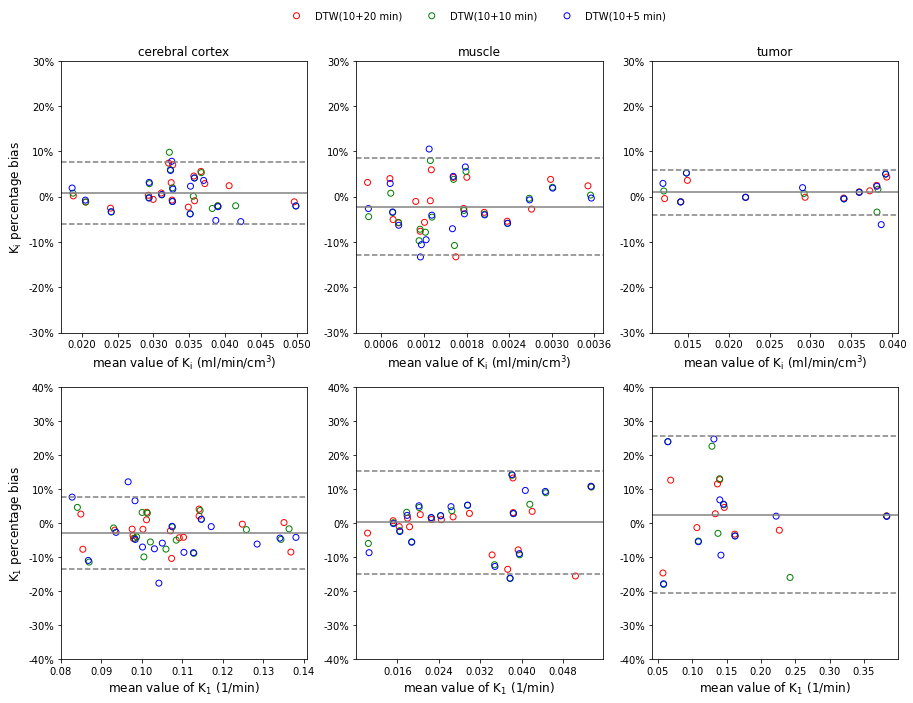


Supplement Figure 5 Bland-Altman plots of the percentage bias in K_i_ and K_1_ derived from the DTW protocol.


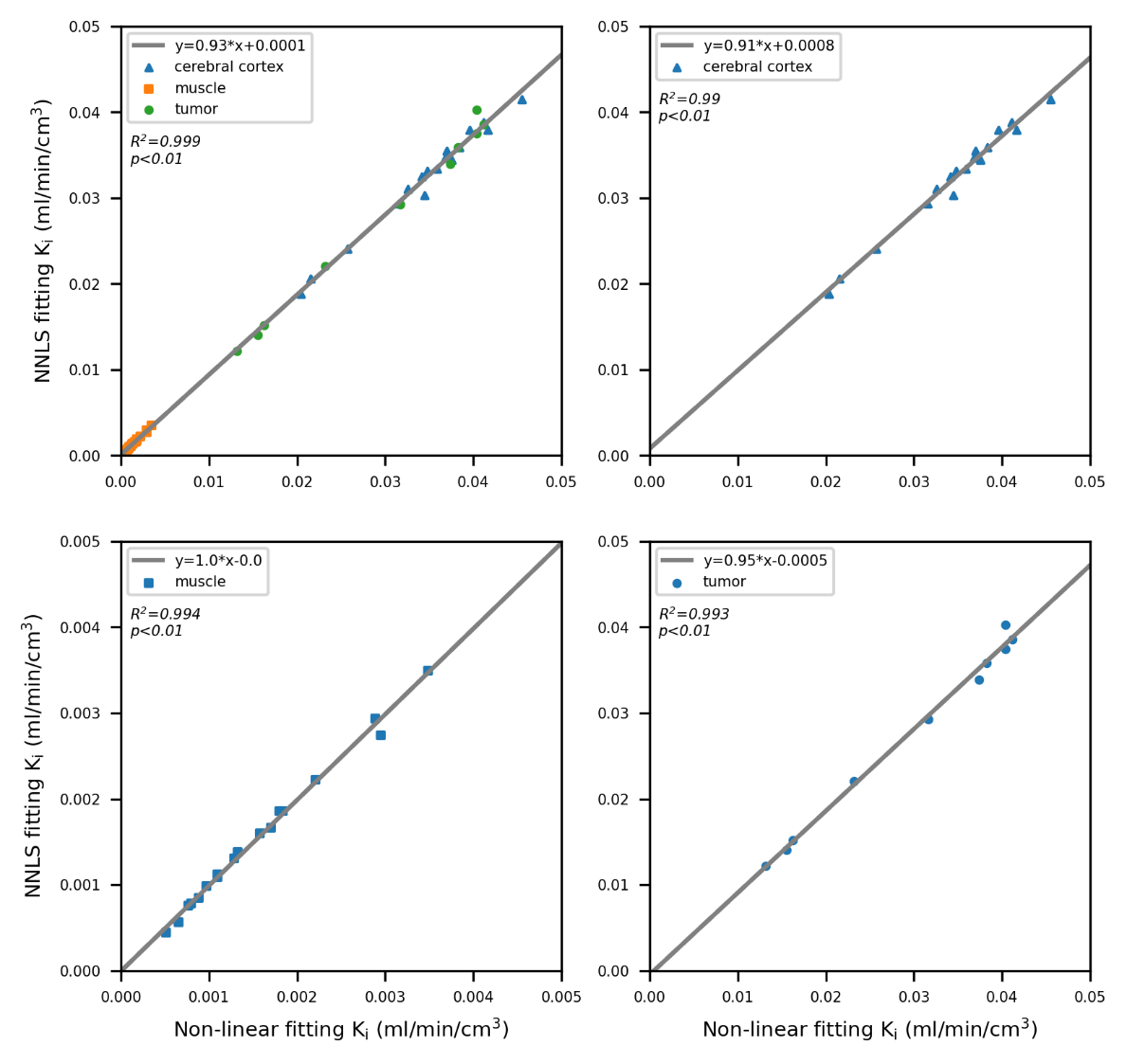


Supplement Figure 6 Correlation analysis between K_i_ estimated by non-linear fitting and Lawson-Hanson NNLS fitting.
